# Cluster analysis of cancer knowledge, attitudes and behaviors in the Moroccan population

**DOI:** 10.1186/s12885-024-12226-5

**Published:** 2024-06-01

**Authors:** Mohamed Khalis, Imad Elbadisy, Oumnia Bouaddi, Amy Luo, Amina Bendriouich, Badr Addahri, Hafida Charaka, Mohamed Chahboune, Jérôme Foucaud, Abdallah Badou, Lahcen Belyamani, Inge Huybrechts

**Affiliations:** 1 Department of Public Health, Mohammed VI Center for Research and Innovation, Rabat, Morocco; 2 Mohammed VI International School of Public Health, Mohammed VI University of Sciences and Health, Casablanca, Morocco; 3 Higher Institute of Nursing Professions and Health Techniques, Rabat, Morocco; 4 Laboratory of Biostatistics, Clinical, and Epidemiological Research, & Laboratory of Community Health (Public Health, Preventive Medicine and Hygiene), Department of Public Health, Faculty of Medicine and Pharmacy, Mohammed V University in Rabat, Rabat, Morocco; 5 Department of Population, Family and Reproductive Health, Johns Hopkins School of Public Health, Baltimore, MD USA; 6 Mohammed VI Faculty of Medicine, Mohammed VI University of Sciences and Health, Casablanca, Morocco; 7 Higher Institute of Health Sciences, Laboratory of Sciences and Health Technologies, Hassan First University of Settat, Settat, Morocco; 8 Institut National du Cancer, Boulogne Billancourt, France; 9 Laboratory of Education and Health Practice, Sorbonne Paris Nord University, Paris, France; 10 Faculty of Medicine and Pharmacy, Hassan II University, Casablanca, Morocco; 11 Faculty of Medicine and Pharmacy, Mohammed V University in Rabat, Rabat, Morocco; 12 Nutrition and Metabolism Branch, International Agency for Research On Cancer, Lyon, France

**Keywords:** Cancer, Morocco, Risk factors, Cluster analysis

## Abstract

**Background:**

Cancer has become a major health concern due to the increasing morbidity and mortality rates, and its negative social, economic consequences and the heavy financial burden incurred by cancer patients. About 40% of cancers are preventable. The aim of this study was to assess the knowledge, attitudes, and practices regarding cancer prevention, and associated characteristics to inform the development of targeted cancer prevention campaigns and policies.

**Methods:**

We conducted a cross-sectional survey of adult patients at Mohamed Sekkat and Sidi Othmane Hospitals in Casablanca, Morocco. Data collection was conducted by two trained interviewers who administered the questionnaire in-person in the local language. An unsupervised clustering approach included 17 candidate variables for the cluster analysis. The variables covered a wide range of characteristics, including demographics, health perceptions and attitudes. Survey answers were calculated to compose qualitative ordinal categories, including a cancer attitude score and knowledge score.

**Results:**

The cluster-based analysis showed that participants in cluster 1 had the highest mean attitude score (13.9 ± 2.15) and percentage of individuals with a high level of knowledge (50.8%) whereas the lowest mean attitude score (9.48 ± 2.02) and knowledge level (7.5%.) were found in cluster 3. The participants with the lowest cancer attitude scores and knowledge levels were aged 34 to 47 years old (middle age group), predominantly females, living in rural settings, and were least likely to report health professionals as a source of health information.

**Conclusions:**

The findings showed that female individuals living in rural settings, belonging to an older age group, who were least likely to use health professionals as an information source had the lowest levels of knowledge and attitudes. These groups are amenable to targeted and tailored interventions aiming to modify their understanding of cancer in order to enhance the outcomes of Morocco’s on-going efforts in cancer prevention and control strategies.

**Supplementary Information:**

The online version contains supplementary material available at 10.1186/s12885-024-12226-5.

## Introduction

Cancer is a leading cause of death and morbidity worldwide [1]. Although the cancer burden has increased globally, the majority (71%) of the 9.9 million cancer deaths in 2020 occurred in low- and middle-income countries (LMICs). By 2040, the increase of cancer burden in LMICs is estimated to be twice that of high-income countries [1]. The rising prominence of cancer as a leading cause of death partly reflects barriers to access or the availability of diagnostic and treatment services alongside population aging and growth. In addition, changing patterns in exposure to cancer risk factors, many of which are associated with socioeconomic development, contributes to the ever-increasing cancer incidence and mortality.

Morocco, like several low- and middle-income countries, faces unique challenges regarding cancer. These challenges include limited access to healthcare services, inadequate infrastructure for cancer diagnosis and treatment, socioeconomic disparities, and specific environmental and lifestyle factors. Due to these challenges, cancer has become a major health concern with increasing morbidity and significant negative social, economic consequences and a heavy financial burden incurred by patients. Data from the cancer registry in the Casablanca region for the 2008–2012 period shows the incidence rate standardized on the global population is 137.3 per 100,000 inhabitants [2, 3]. According to the Global Cancer Observatory, the age standardized incidence rate for cancer in Morocco was 148.3 per 100,000 inhabitants and the ASR mortality rate was 87.9, as of 2020 [3]. The risk of developing cancer is significantly linked with lifestyle choices, which are notably determined by the social environment. There are several uncontrollable risk factors, but it is possible to modify lifestyle and take action on the environmental level to reduce the increased threat of cancer [4]. In fact, over 40% of cancers may be preventable by targeting key risk factors such as diet, physical activity and tobacco and alcohol consumption, as well as taking recommended vaccines and addressing environmental pollution [5, 6].

Cognizant of this growing burden, cancer control and prevention have occupied a priority place in Morocco’s health agenda. In 2010, Morocco became the first North African country to operationalize a National Cancer Control Plan. Guided by the plan’s strategic measures, the Ministry of Health and Social Protection with the support of Non-Governmental Organizations and partners such as the Lalla Salma Foundation for Cancer Prevention and Treatment (LSFCPT), have made significant investments in improving diagnostic and therapeutic services by increasing the number of specialized health facilities and increasing the number of trained and specialized healthcare providers [4, 5]. In addition, the National Cancer Control Plan has placed considerable importance on primary prevention. Within these measures, community education campaigns via schools, work places, and mass media have encouraged individuals to adopt preventative activities addressing cancer risk factors [2, 7, 8]. Furthermore, intensified efforts have been undertaken to address the most prevalent types of cancers such as breast and lung cancer, through mass screening campaigns and public awareness campaigns addressing main risk factors such as tobacco and excessive alcohol consumption. Despite substantial progress on these fronts, a substantial portion of the cancer morbidity and mortality in Morocco is preventable. Of the most common cancers, lung and cervical cancer are amenable to primary prevention, and mortality from breast, colorectal, and cervical cancer can be substantially reduced by early detection and effective treatment. However, a majority of cancers, are diagnosed at advances stages, where curative therapies are less effective [4]. It is well known that protective behavior can prevent many cancers, and knowledge is a prerequisite for such behavioral change [9]. In fact, it was shown in the 1960s that a good level of knowledge/adequate attitude made it possible to promote the development of health-protective environments and behaviors [10].

While a number of studies have explored knowledge, attitudes, and practices [11] and associated characteristics, the majority of studies examine specific cancers, such as breast, cervical, and skin cancers [8, 9, 12–15]. Only one study, conducted before the implementation of the National Cancer Control Plan, comprehensively examined the awareness of the main cancer risk factors in the general population [14], despite the relevance of this information to the development of comprehensive cancer control plan. The success of cancer treatment is dependent on disease stage and the timeliness of diagnosis and treatment. Often, symptoms develop before the disease progresses, and in the case of Morocco, relatively large-scale screening programs are only available for the most prevalent cancer types. Therefore, in most cases, symptoms should be recognized by patients and brought to the attention of physicians. In this sense, exploring attitudes and knowledge among the general population can provide insights into the likelihood of recognizing early symptoms and seeking care, thereby improving chances of survival. Therefore, in this study, we aim to assess the knowledge, attitudes, and practices regarding cancer prevention, and associated characteristics to inform the development of targeted cancer prevention campaigns and policies.

## Methods

### Study design

We conducted a cross-sectional survey of patients at Mohamed Sekkat and Sidi Othmane Hospitals in Casablanca, Morocco between September 2021 and February 2022.

### Study population

Participants were randomly selected upon admittance to department of outpatient consultation. This department provides health consultations to patients for various medical and surgical specialties and receives patients from the general population with different socio-demographic background. Participants under 18 years of age, currently hospitalized, or with a history of cancer were excluded.

The sample size was calculated using OpenEpi software and the following formula:

$$\mathrm n\;=\;\lbrack\mathrm{DEFF}\ast\mathrm{Np}(1-\mathrm p)\rbrack/\;\lbrack(\mathrm d2/\mathrm Z21-\mathrm\alpha/2\ast(\mathrm N-1)+\mathrm p\ast(1-\mathrm p)\rbrack$$

Where N is the population size (for the finite population correction factor or fpc) = 8000; p is the hypothetical frequency (%) of the outcome factor in the population (50% ± 5; % confidence limits of 100) (absolute ± %)(d) = 5%; DEFF (design effect = 1); Z is a constant = 1.96. for a 95% confidence interval. Based on the above parameters, the minimum required sample size (n) was 734 participants across both centers.

### Data collection

Data collection was conducted by two trained interviewers who administered the questionnaire in-person in the local language (French). The data collection questionnaire was developed based on a review of relevant literature and other similar studies [16, 17]. The questionnaire was tested through a pilot study involving a group of 10 randomly selected patients at the outpatient centers. The data collected during this pilot phase were reviewed for consistency and reliability. In addition, feedback from the pilot was used to make necessary adjustments to the questionnaire before the full study was conducted. The questionnaire comprised four sections: demographic characteristics, knowledge of cancer risk factors, perceptions and attitudes towards cancer, and individual practices and exposure to risky behaviors. Knowledge was assessed using 22 items. Of these, 19 question items asked respondents to rate their level of certainty (e.g. “certainly”, “probably”, “certainly not”, “I don’t know”); with regards to a list of possible causes of cancer these included; tobacco and alcohol use, unprotected sun exposure, sedentary lifestyle, overweight, unhealthy diet, air pollution, stressful life events, excessive radiation, contraceptive use, use of hormone replacement therapy, and exposure to infectious pathogens such as HPV and EBV. Knowledge was also assessed regarding cancer risk in relation to frequent consumption of seven food items; fruits and vegetables, red meat, white meat, deli meats, fish, salt and salted food, and sugar. Participants’ attitudes towards cancer were assessed based on their agreement with a list of 6 items; contagion, heredity, perceived cancer risk, perceived capability to prevent cancer, disclosing cancer diagnosis to others, ability of cancer patients to lead a normal life; using a four-point Likert scale (e.g. “strongly agree”, “tend to agree”, “tend to disagree”, “strongly disagree”). Behaviors were assessed using 10 items on self-reported health status and behaviors. The cancer risk factors examined included tobacco use, alcohol consumption, unprotected sun exposure, and diet. To minimize respondent bias, participants were assured of anonymity and confidentiality, questions were sensitively crafted, interviews conducted in comfortable settings, and the questionnaire pilot-tested for comprehension and sensitivity. The questionnaire is given in Supplementary material.

### Statistical analysis

Data analysis was performed using R software. Descriptive analyses were performed using frequencies (percentages) for categorical variables and means (± standard deviation) for continuous variables. A one-way ANOVA test was used for mean comparison (more than 2 samples) and chi-squared test was used for proportion comparison.

An unsupervised clustering approach included 17 candidate variables for the cluster analysis. The variables covered a wide range of characteristics, including demographics, health perceptions and attitudes. Survey answers were calculated to compose qualitative ordinal categories, including a cancer attitude score and knowledge score. The knowledge score was calculated based on 22 items scored on a Likert scale (“certainly” = 3, “probably” = 2, “certainly not” = 1, “I don’t know” = 0). The total score was calculated by summing the points corresponding to the items for each individual. The knowledge score ranged from 0 to 66 before finally converting it to a categorical variable using tertiles (tertile 1 = High; tertile 2 = Medium; tertile 3 = Low). The attitude score was calculated based on 6 items scored on a Likert scale (“strongly agree” = 3, “tend to agree” = 2, “tend to disagree” = 1, “strongly disagree = 0”). Consequently, the attitude score ranged from 0 to 18.

The method used to build the clusters is based on the k-prototypes algorithm [18]. his is an unsupervised learning algorithm used for clustering mixed type data. This method seems to perform well with heterogeneous data [19]. The choice of the k-prototypes algorithm for our dataset was driven by the characteristics of the data collected from the cross-sectional survey. This dataset comprises a mix of categorical and numerical variables, reflecting a wide range of characteristics such as demographics, health perception, attitudes, and addictions. The k-prototypes algorithm is an extension of the k-means paradigm that is specifically designed to cluster data with mixed types. It combines the k-means' approach for numerical attributes with the k-modes approach for categorical attributes, using a cost function that accommodates both attribute types. This makes the k-prototypes algorithm particularly suitable for our dataset, as it allows us to cluster individuals based on a comprehensive set of variables without losing the integrity of the categorical data.

The algorithm defines virtual individuals or prototypes as cluster centers using group means for numerical variables and modes for categorical variables. For this, two distance metrics are used: Euclidean distance for the continuous variable and the Hamming distance [20]. In practice, between 2 individuals the distance is defined as follows:

$${d}_{2}\left(X,Y\right)=\sum {\left({x}_{j}-{y}_{j}\right)}^{2}+\gamma \sum \delta \left({x}_{j},{y}_{j}\right)$$

The first term of the equation corresponds to the squared Euclidean distance of the continuous variables while the second term correspond the Hamming distance of the categorical variables. The minimization criteria are the total sum of distances between individuals and the prototype of the cluster ${b}_{g}$ to which they belong:

$$TSD=\sum \sum \left(\sum {\left({x}_{j}-{b}_{g}{,}_{j}\right)}^{2}\right)+\gamma \sum \delta \left({x}_{j},{b}_{g}{,}_{j}\right)$$

The k-prototypes algorithm is very similar to k-means: the initial G-prototypes are selected as cluster centers temporarily, and then each individual is matched to the nearest center. An iteration of the allocation process is performed until the most optimal allocation is obtained. To determine the driving variables that were most involved in the construction of the clusters, we used an innovative method based on feature importance permutation principle [21]. The selection of the k-prototypes algorithm was driven by its ability to adeptly handle our dataset's mixed data types, leading to the identification of 3 optimal clusters through the silhouette and Elbow methods. The validation of our clusters involved silhouette analysis to ensure internal consistency, and hypothesis testing to confirm significant differences between clusters, establishing a strong statistical foundation for our findings. The importance of a given variable is defined by the decrease in the model score when the variable is randomly shuffled. The magnitude of the decrease indicates how much the model depends on the variable. The analysis was carried out using R software [22]. The clustering algorithm used is implemented in the clustMixType package [23]. The feature ranking procedure is implemented in FeatureImpClust [24].

To address the challenge of missing data in our dataset, we first analyzed the distribution of missingness—particularly, professional status (15% of missing values), BMI category (8% of missing values), and Cancer knowledge score (1%). This comprehensive analysis helped us understand the patterns and extents of missingness across different variables. For the imputation of missing data, we employed the missRanger package, an innovative machine learning-based algorithm well-suited for this task [24]. The missRanger algorithm leverages the strengths of random forests combined with predictive mean matching to impute missing values. This combined approach allows for the replacement of missing values with plausible values from similar cases, enhancing the plausibility of the imputed data with high predictive accuracy imputation.

### Ethical considerations

The study protocol was approved by the Ethics Committee of Cheikh Khalifa Hospital of Casablanca (CE_UM6SS/26/03/2021). Informed consent was obtained from all participants prior to the study. Participation in this study was voluntary and was not compensated. All aspects of this study including design and implementation were carried out in accordance with the ethical principles outlined in the Helsinki Declaration.

## Results

### General characteristics

A total of 743 participants were included in this study. By age group, 248 (33.4%), 249 (33.5%), and 246 (33.1%) of participants were aged 18–33, 34–47, and 48–85, respectively (Table 1, Overall column). The majority of participants (60.2%) were female and lived in an urban area (71.5%). Overall, the knowledge levels were nearly almost distributed among participants; 36.1% of participants had a low level of knowledge, 30.6% of participants had a medium level, and 33.4% of participants had a high level of cancer knowledge. The overall attitude score was 11.9 ± 3.02.

**Table 1:** Knowledge, attitudes, and associated characteristics among clusters of respondents

	**1** **(*N* = 244)** **n (%)**	**2** **(*N* = 258)** **n (%)**	**3** **(*N* = 241)** **n (%)**	**Overall** **(*N* = 743)** **n (%)**	***P*-value**
**Age**					
18–33	46 (18.9)	126 (48.8)	76 (31.5)	248 (33.4)	< 0.001
34–47	66 (27.0)	88 (34.1)	95 (39.4)	249 (33.5)	
48–85	132 (54.1)	44 (17.1)	70 (29.0)	246 (33.1)	
**Sex**					
Female	198 (81.1)	63 (24.4)	186 (77.2)	447 (60.2)	< 0.001
Male	46 (18.9)	195 (75.6)	55 (22.8)	296 (39.8)	
**Marital status**					
Divorced	16 (6.6)	17 (6.6)	20 (8.3)	53 (7.1)	< 0.001
Married	155 (63.5)	151 (58.5)	139 (57.7)	445 (59.9)	
Single	52 (21.3)	86 (33.3)	44 (18.3)	182 (24.5)	
Widowed	21 (8.6)	4 (1.6)	38 (15.8)	63 (8.5)	
**Professional status**					
Active	45 (18.4)	186 (72.1)	46 (19.1)	277 (37.3)	< 0.001
Non active	199 (81.6)	72 (27.9)	195 (80.9)	466 (62.7)	
**Place of residence**					
Rural	5 (2.0)	41 (15.9)	166 (68.9)	212 (28.5)	< 0.001
Urban	239 (98.0)	217 (84.1)	75 (31.1)	531 (71.5)	
**BMI categories**					
Normal	71 (29.1)	151 (58.5)	79 (32.8)	301 (40.5)	< 0.001
Overweight/Obese	167 (68.4)	101 (39.1)	156 (64.7)	424 (57.1)	
Underweight	6 (2.5)	6 (2.3)	6 (2.5)	18 (2.4)	
**Tobacco use**					
No	227 (93.0)	147 (57.0)	189 (78.4)	563 (75.8)	< 0.001
Yes	17 (7.0)	111 (43.0)	52 (21.6)	180 (24.2)	
**Alcohol consumption**					
No	238 (97.5)	195 (75.6)	194 (80.5)	627 (84.4)	< 0.001
Yes	6 (2.5)	63 (24.4)	47 (19.5)	116 (15.6)	
**Passive smoking**					
No	162 (66.4)	49 (19.0)	140 (58.1)	351 (47.2)	< 0.001
Yes	82 (33.6)	209 (81.0)	101 (41.9)	392 (52.8)	
**Health conditions**					
No	76 (31.1)	209 (81.0)	185 (76.8)	470 (63.3)	< 0.001
Yes	168 (68.9)	49 (19.0)	56 (23.2)	273 (36.7)	
**Reported health status**					
Good	143 (58.6)	190 (73.6)	177 (73.4)	510 (68.6)	< 0.001
Poor	69 (28.3)	19 (7.4)	41 (17.0)	129 (17.4)	
Very good	32 (13.1)	49 (19.0)	23 (9.5)	104 (14.0)	
**History of cancer**					
No	92 (37.7)	81 (31.4)	50 (20.7)	223 (30.0)	0.001
Yes	152 (62.3)	177 (68.6)	191 (79.3)	520 (70.0)	
**Skin examination**					
No	177 (72.5)	205 (79.5)	220 (91.3)	602 (81.0)	< 0.001
Yes	67 (27.5)	53 (20.5)	21 (8.7)	141 (19.0)	
**Health professionals as information source**					
Non	145 (59.4)	190 (73.6)	210 (87.1)	545 (73.4)	< 0.001
Yes	99 (40.6)	68 (26.4)	31 (12.9)	198 (26.6)	
**Attitudes score**					
Mean (SD)	13.9 (2.15)	12.3 (2.92)	9.48 (2.02)	11.9 (3.02)	< 0.001
Median [Min, Max]	14.0 [6.00, 18.0]	12.0 [3.00, 18.0]	10.0 [3.00, 18.0]	12.0 [3.00, 18.0]	
**Level of knowledge**					
High	124 (50.8)	106 (41.1)	18 (7.5)	248 (33.4)	< 0.001
Low	38 (15.6)	63 (24.4)	167 (69.3)	268 (36.1)	
Medium	82 (33.6)	89 (34.5)	56 (23.2)	227 (30.6)	

### Description and comparison of clusters

The cluster-based analysis showed that participants in cluster 1 had the highest mean attitudes score (13.9 ± 2.15) and percentage of individuals with a high level of knowledge (50.8%) compared to other clusters, and this difference was statistically significant (*p* < 0.001) (Table 1). The majority of individuals in cluster 1 were aged 48 years and older (54.1%), female (81.1%), married (63.5%), living in an urban area (98%), overweight or obese (68.4%), presenting with a health a condition (68.9%), had a history of cancer (62.3%) and no reported use of tobacco (93%) or alcohol consumption (97.5%). Compared to other clusters, cluster 1 also had the highest percentage of individuals who use health professionals as their main information source (40.6%, *p* < 0.001) and the highest percentage of individuals who have undergone skin examination (27.5%, *p* < 0.001).

Cluster 3 had the lowest mean attitudes score of (9.48 ± 2.02) and the lowest percentage of individuals with a high level of knowledge (7.5%) compared to clusters 1 and 2 (*p* < 0.001). Most individuals in cluster 3 were female (60.2%), aged 34 to 47 years (39.4%) living in a rural area (68.9%), overweight/obese (64.7%), with no pre-existing health conditions (23.2%), and no reported use of tobacco (78.4%) or alcohol consumption (80.5%). Compared to other clusters, cluster 3 had the lowest percentage of individuals who use health professionals as their main information source (12.9%, *p* < 0.001), and the highest percentage of individuals with a history of cancer (79.3%, *p* < 0.001).

In cluster 2, the mean attitude score was 12.3 ± 2.92 and the percentage of individuals with a high level of knowledge was 41.1%. These values are significantly higher compared to cluster 3 and significantly lower compared to cluster 1 (*p* < 0.001). Compared to clusters 1 and 3, cluster 2 participants were younger (48.8% aged 18 to 33, *p* < 0.001), predominantly male (75.6%, *p* < 0.001), mostly healthy (81%, *p* < 0.001) and had a normal BMI (58.5%, *p* < 0.001). This cluster recorded the highest rates of tobacco use (43%), alcohol consumption (24.4%) and passive smoking (81%) compared to the remaining clusters (*p* < 0.001).

## Discussion

The aim of this study was to assess knowledge, attitudes, and behaviors regarding cancer prevention, and describe the associated characteristics using a cluster analysis approach. We identified 3 participant profiles of greatest to lowest cancer attitude scores and cancer knowledge levels. Profiles were defined by the 14 variables that examined sociodemographic characteristics as well as health history and behaviors. Overall, the participants with the lowest cancer attitude scores and knowledge levels were aged 34 to 47 years, predominantly female, living in rural settings and were least likely to report health professionals as a source of health information. This is aligned with previous studies that have examined breast and cervical cancer awareness and practices in Morocco, where factors such as age, employment, marital status, residence, smoking status, and risky health behaviors were associated with knowledge among Moroccan women [8, 9, 12, 15].

This study found level of knowledge about cancer and cancer prevention. Other studies in LMICs showed poor levels of public knowledge about cancer symptoms (67.6%) in Saudi Arabia and behavioral risk factors (61%) in Ethiopia [25, 26]. Similarly, another study in Turkey revealed that adult individuals had a significant level of false and incomplete information about cancer [27].

This study reported that living in urban areas was significantly associated with a high knowledge score in the respect of cancer risk factors which is consistent with the findings of a previous study on non-specific cancer risk factors in Morocco [15]. Other studies examining knowledge related to specific types of cancer also reported a link between rural residence and lower knowledge scores [15]. This may be explained by the centralization of services and health facilities in urban areas which not only results in a lack of information around risk factors but also leads to significant delays in diagnosis and the initiation of treatment [15, 28].

In this study, knowledge scores were found to be higher among participants with no history of tobacco or alcohol consumption. Similar findings were reported in a previous study in Morocco where smoking and alcohol consumption were associated with of knowledge levels [15], while contrasted results were reported in France where higher knowledge was found among smokers compared to non-smokers. Tobacco use is pervasive in LMICs and is a common risk factors in malignancies. In Morocco, the prevalence of tobacco use was 18% in 2018. In this study, a significantly high prevalence of tobacco use (43%) and passive smoking (81%) was reported among the relatively young cluster, which reflects the trends in tobacco consumption in the general population. Although the prevalence of alcohol consumption in Morocco is low due to restrictive policies, alcohol consumption was found to be significantly high in the younger cluster (24.4%) in this study. This calls for intensified efforts to increase awareness about the harmful effects of alcohol consumption particularly in younger age groups who are often at-risk of adopting such unhealthy behaviors thereby undermining their health outcomes in adulthood.

The use of health professionals as a source of knowledge was higher in clusters with high knowledge levels; however, this percentage remains relatively low (below 50%) for all clusters. This may be due to family members and close environment being the most trusted sources of information as reported by previous studies [14]. This may also be explained by the lack of information among healthcare professionals about cancer risk factors and preventive practices. In fact, several studies assessed knowledge of health professionals on specific-cancer risks and have reported insufficient or unsatisfactory levels of knowledge, particularly among general practitioners, even regarding prevalent types such as breast and cervical cancer [29–32]. In fact, one study in Beni-Mellal province found that almost half of general practitioners (GPs) (49.3%) were unaware of the existence of a national cancer prevention and control plan in Morocco [31]. Moreover, awareness of cancer risk and prevention was reported to be higher among physicians in urban areas which may explain the differences in knowledge levels between rural and mostly urban- clusters in this study [31]. GPs play an important role in raising awareness about cancer risk and increasing adherence to preventive behaviors such as screening. For instance, advice from a healthcare professional to stop smoking has been shown to increase the six-month cessation rate by around 70%, however large-scale cancer research in France showed that only 23% of smokers have discussed smoking with a healthcare professional in the last 12 months, of which 15% was initiated by patients and only 8% were initiated by health professionals [33, 34]. Thus, the need to promote continuous education and adequate training in the field of cancer prevention is detrimental to the success of on-going prevention efforts including major screening programs in Morocco such as breast and cervical cancer.

**Fig. 1:**
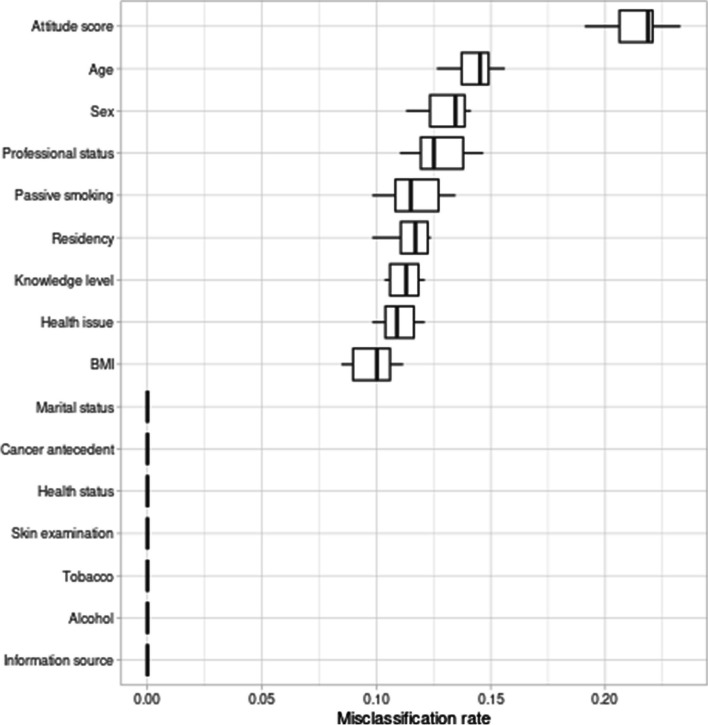
Feature importance in clusters formation

Understanding the importance of features in cluster construction is an important aspect of data analysis. The importance of this feature is often quantified by measuring the misclassification rate relative to the baseline cluster assignment, which is derived from a random permutation of feature values. The significance of this approach becomes evident when we consider that, in the majority of cluster analysis-based studies, such vital information is not typically provided. As illustrated in Fig. 1, it becomes clear that the " Attitude Score " feature plays a pivotal role in constructing our clusters, followed closely by "Age," "Sex," and "Professional Status." On the other hand, features such as "Marital Status," "Cancer History," and "Information Source," among others, have a less pronounced impact on cluster formation. This knowledge serves as a valuable tool, especially in the context of preventive public health policies, where prioritizing prevention efforts is essential. By directly targeting these key drivers, we can implement effective policies to address and mitigate the identified factors, ultimately leading to more successful prevention strategies.

Awareness of cancer risk factors and early symptoms plays a pivotal role in prevention and early detection of cancer and influences the time to presentation for diagnosis. In addition, adequate knowledge about cancer prevention and treatment is a pre-requisite to taking individual preventive actions and reducing unhealthy lifestyle behaviors in relation to cancer [4]. Identifying subgroups with low levels of KAP is an opportunity to implement precise and tailored interventions to improve knowledge and uptake of prevention and care services thereby bolstering the efficacy of Morocco's ongoing initiatives in cancer prevention and control. Effective efforts to expand education and outreach campaigns should focus on and address the needs of the least informed groups potentially, at a higher risk of developing cancer. For instance, creating separate education programs to target specific population groups can be guided by the profiles described here. These include females, living in rural settings, belonging to an older age group, and people who are least likely to use health professionals as an information source. These groups that may benefit most from tailored cancer awareness campaigns should be targeted given resource constraints. Potential strategies include reaching audiences where health professionals are not considered a health information source and may engage in more health harming behaviors such as tobacco and alcohol consumption. Other avenues for disseminating information and facilitating education should be explored for these groups, including engagement with community and religious leaders. It is also important to recognize the diversity within Moroccan culture, characterized by a multitude of dialects including Tamazight and Darija. This diversity emphasizes the importance tailoring language and communication to accommodate different linguistic and cultural differences and to make sure that the information resonates with the different segments of the Moroccan population.

It is worthy of note that while an individual's level of education and awareness are key in shaping knowledge and attitudes related to cancer, it is important to acknowledge the broader social, political, economic, and commercial factors that might influence KAP. Governments, industries, regulations, and media play significant roles in creating an environment conducive to healthy choices and behaviors. For instance, industries such as tobacco and alcohol hold responsibility and can significantly impact KAP related to cancer, as seen in instances where they misinterpret evidence and disseminate misleading health information regarding the impact of their products on cancer risk [35]. Policymakers, academics, public health professionals, and other practitioners should reassess the appropriateness of their relationships with these industries to promote unbiased public awareness about cancer and its risk factors [35, 36].

This study has some limitations. Our sampling was restricted to subjects attending only two healthcare facilities in Casablanca; therefore, the extrapolation of our findings to the general population should be interpreted with caution. To the best of our knowledge, this is the first study to use cluster-analysis to assess non-specific cancer knowledge, attitudes and behaviors. The findings in this study provide new evidence of gaps in cancer awareness in the general population. Each of the participant profiles inform areas for improvement in policy and practice for cancer knowledge, attitudes, and practices.

## Conclusions

This study identified profiles in the general population in Morocco with variable levels of knowledge and attitudes towards cancer prevention. The findings showed that female individuals living in rural settings, belonging to an older age group, who were least likely to use health professionals as an information source had the lowest levels of knowledge and attitudes. These groups are amenable to targeted and tailored interventions aiming to modify their understanding of cancer in order to enhance the outcomes of Morocco’s on-going efforts in cancer prevention and control strategies. These groups should be the primary focus of preventive interventions and screening. To validate the results of this study and extend their applicability to all Moroccan population, a larger, multicenter study is necessary.

### Supplementary Information

**Supplementary Material 1.**

## Data Availability

Data are available upon reasonable request from the corresponding author.

## Abbreviations

LMICs: Low and middle-income countries
LSFCPT: Lalla Salma Foundation for Cancer Prevention and Treatment
