# Supplementary material for: Cluster analysis of cancer knowledge, attitudes and behaviors in the Moroccan population
Source: BMC Cancer. 2024 Jun 1;24:669. doi: 10.1186/s12885-024-12226-5 (PMC11143602; doi:10.1186/s12885-024-12226-5)
Supplement: Supplementary file 1 — Supplementary Material 1. [file 12885_2024_12226_MOESM1_ESM.docx]

**Supplementary file : questionnaire**

**Participant number :**

|__||__||__||__|

**SECTION 1 : GENERAL INFORMATION**

**Q1. Age**: |__||__|

**Q2. Gender:**

1. Male

2. Female

**Q3. Place of residence :**

1. Urban

2. Rural

**Q4. Marital status :**

1. Single

2. Married

3. Divorced

4. Widowed

5. Other, please specify : ___________

**Q5. What is your current situation ?**

1. Employed

2. Retired

3. Unemployed/seeking first employment

6. Student

8. Not enrolled in school

9. Housewife

**Q6. Can you read and write ?**

1. Yes
2. No

**Q7. What is the highest level of education you have achieved ?**

1. None

2. Quranic school

3. Primary school

4. Secondary school

5. Vocational education (in high school, college)

6. University

**Q8. What is your height ? (cm)** |__||__||__|

**Q9. What is your weight ? (kg)** |__||__||__|

(NB : If pregnant, please specify the weight before pregnancy)

**Q10. Have you, or have you had, among your relatives (family, friends, colleagues), a person affected by cancer?**

1. Yes

2. No

**Q11. If yes :**

1. In your family

2. Among your friends

3. Among your colleagues

4. Among other acquaintances

**SECTION 2 : HEALTH STATUS**

**Q12. How would you describe your overall health?**

1. Very good

2. Good

3. Fairly good

4. Bad

5. Very bad

**Q13. Do you have an illness or health problem that is chronic or has lasted at least 6 months ?**

1. Yes

2. No

**SECTION 3 : OPINION**

**Q14. Which three diseases do you consider the most severe ?**

1. Cancer
2. Diabetes
3. COVID-19
4. Seasonal flu (ex: H1N1, …)
5. Hepatitis B
6. Hepatitis C
7. HIV/AIDS
8. Tuberculosis
9. Alzheimer’s disease
10. Heart disease/heart attack
11. Brain stroke
12. Other, please specify ……………………………

**Q15. In your opinion, what are the three most frequent cancer sites in Morocco ?**

1. Cervix

2. Colon, rectum

3. Prostate

4. Breast

5. Skin

6. Lung

7. Pancreas

8. Leukemia

9. Other, please specify …………………………………

**SECTION 4 : HEALTH PERCEPTION AND INFORMATION SOURCES**

**Q16. In general, are you aware of any physical changes that would make you think you have cancer?**

1. Yes

2. No

**Q 17. If yes, could you name three of them ?**

1. ……………………………………

2. ……………………………………

3. ……………………………………

**Q18. If you notice a physical sign that raises concerns about the possibility of having cancer, what actions would you take?**

1. Speak to a medical professional first

2. Speak to a relative

3. Look for the information by yourself

4. Wait and see if the symptom disappears

5. Other, please specify ……..

**Q19. What is your main source of information about cancer ?**

1. TV/Radio
2. Health journals/magazines
3. Social media (Facebook, WhatsApp…)
4. Health professionals (pharmacist, doctor, nurse…)
5. Your family
6. Professional circle
7. Book/reading
8. Prevention campaigns
9. Associations
10. Other, please specify………………………….………

**SECTION 5 : ALCOHOL AND TOBACCO**

**Q20. In the past twelve months, have you ever consumed alcoholic beverages?**

1. Yes

2. No

**Q21. Have you ever tried smoking tobacco (including e-cigarettes) in your lifetime ?**

1. Yes

2. No

**Q22. If yes, how many cigarettes did you smoke on average per day?**  |__||__|

**Q23. Are you currently exposed to tobacco smoke (passive smoking)?**

1. Never, or almost never
2. Less than one hour per day
3. 1 à 2 heures par jour
4. 1 to 2 hours per day
5. More than 5 hours per day

**SECTION 6 : SUN EXPOSURE**

**Q24. What measures do you take to protect yourself from the sun on a bright summer day ?**

1. Avoid the sunniest hours between 12 PM and 4 PM
2. Stay in the shade
3. Applying sunscreen every two hours
4. Wearing sunglasses
5. Wearing a hat or a cap
6. Wear long clothes

**Q25. Does your job require you to work in the sun ?**

1. Yes, systematically

2. Yes, regularly

3. Yes, occasionally

4. No, never

**Q26. Do you ever have your skin examined by a doctor to check for anomalies, such as appearing or changing moles ?**

1. Yes, it happened to me
2. Yes, every year
3. No, It never happened to me
4. I don’t remember

**Q27. Do you think a mole can turn into cancer ?**

1. Yes
2. No
3. I don’t know

**SECTION 7 : NUTRITION AND BREASTFEEDING :**

**Q28. In your opinion, breastfeeding :**

1. Can decrease the risk of breast cancer in the mother
2. Can increase the risk of breast cancer in the mother
3. Does not have in impact on the risk of breast cancer in the mother

**Q29. Do you believe that diet plays a crucial role in the development of cancer?**

1. Very important
2. Somewhat important
3. Not particularly significant
4. Not significant at all

**Q30. According to you, frequent consumption of:**

|  | **Can decrease the risk of cancer** | **Can increase the risk of cancer** | **Has no impact on the risk of cancer** | **I don’t know** |
| --- | --- | --- | --- | --- |
| **1. Fruits and vegetables** |  |  |  |  |
| **2. Red meat** |  |  |  |  |
| **3. Processed meat** |  |  |  |  |
| **4. White meat** |  |  |  |  |
| **5. Fish** |  |  |  |  |
| **6. Salt and salty foods** |  |  |  |  |
| **7. Sugar** |  |  |  |  |

**SECTION 8 : KNOWLEDGE OF RISK FACTORS**

**Q31. Now let’s discuss potential factors that can potentially contribute to the development of cancer. I will mention various behaviors, and for each one, please indicate whether you believe it has a significant influence on the development of cancer**

|  | **Definitely** | **Probably** | **Definitely not** | **I don’t know** |
| --- | --- | --- | --- | --- |
| **1. Smoking tobacco** |  |  |  |  |
| **2. Regular alcohol consumption** |  |  |  |  |
| **3. Unprotected sun exposure** |  |  |  |  |
| **4. Sedentary lifestyle and lack of physical activity** |  |  |  |  |
| **5. Being overweight (BMI> 25 kg/m²) or obese (BMI> 30 kg/m²) in adulthood** |  |  |  |  |
| **6. Consuming foods treated with chemicals (colorants, preservatives, pesticides…)** |  |  |  |  |
| **7. Breathing polluted air (vehicles exhaust, factory fumes, waste incinerators…)** |  |  |  |  |
| **8. Living near a mobile phone relay antenna** |  |  |  |  |
| **9. Enduring the stress of modern life** |  |  |  |  |
| **10. Being emotionally affected by painful experiences such as bereavements, separations or unemployment** |  |  |  |  |
| **11. Being exposed to chemical substances (endocrine disruptors, heavy metals, pesticides, nanomaterials, organic compounds…) in one’s occupational activity** |  |  |  |  |
| **12. Undergoing numerous diagnostic imaging examinations (MRI, CT Scan)** |  |  |  |  |
| **13. Taking contraceptives** |  |  |  |  |
| **14. Hormone replacement therapy for menopausal women** |  |  |  |  |
| **15. Exposure to certain infectious agents, viruses or bacteria (HPV, EBV, Helicobacter pylori, hepatitis virus…)** |  |  |  |  |
| **16. Working night shifts or on rotating schedules** |  |  |  |  |
| **17. Risky sexual behavior** |  |  |  |  |

**SECTION 9 : ATTITUDES AND PERCEPTIONS**

**Q32. I will now present you with opinions about cancer in general. Please indicate, for each of them, whether you strongly agree, somewhat agree, somewhat disagree, or strongly disagree.**

|  | **Strongly agree** | **Somewhat agree** | **Somewhat disagree** | **Strongly disagree** |
| --- | --- | --- | --- | --- |
| **1. Some types of cancer are contagious** |  |  |  |  |
| **2. Cancer is often hereditary** |  |  |  |  |
| **3. Nobody is immune to cancer** |  |  |  |  |
| **4. Cancer is unpreventable** |  |  |  |  |
| **5. It is recommended to have as much open discussions as possible about cancer with relatives when diagnosed with it** |  |  |  |  |
| **6. When someone has cancer, they are often sidelined or excluded** |  |  |  |  |
| **7. When someone has cancer, they are no longer able to work as they did before their diagnosis** |  |  |  |  |
| **8. Cancer is a disease like any other** |  |  |  |  |
| **9. After experiencing cancer, individuals can still lead a normal life** |  |  |  |  |
